# Glucans with Different Degrees of Polymerization from *Leuconostoc mesenteroides* CICC6055: Analysis of Physicochemical Properties and Intestinal Prebiotic Function

**DOI:** 10.3390/ijms25010258

**Published:** 2023-12-23

**Authors:** Jiabao Gu, Ziyan Jiao, Tao Wang, Bolin Zhang, Hongfei Zhao

**Affiliations:** Beijing Key Laboratory of Forest Food Processing and Safety, College of Biological Science & Biotechnology, Beijing Forestry University, Beijing 100083, China; gjb9959228@163.com (J.G.); zyann0403@163.com (Z.J.); wtshro@sina.com (T.W.); zhangbolin888@163.com (B.Z.)

**Keywords:** *Leuconostoc mesenteroides* CICC6055, oligoglucan, gut microbiota, in vitro simulated fermentation

## Abstract

This study explored the physicochemical properties and prebiotic activities of glucans and oligoglucans. Oligoglucans were obtained through the fermentation of *Leuconostoc mesenteroides* CICC6055 and the glucansucrase of strain CICC6055, while glucans were obtained only through fermentation. Thin-layer chromatography and high-performance liquid chromatography identified enzymatically synthesized oligoglucans with a higher yield. Differential scanning calorimetry and derivative thermogravimetry analyses revealed the heat resistance of the glucans and oligoglucans at 280–300 °C. Fourier transform-infrared spectroscopy and nuclear magnetic resonance analyses demonstrated that their main chains were linked with α-1,6-glycosidic bonds accompanied by glucose residue branching. In vitro fermentation experiments demonstrated that they not only improved the contents of short-chain fatty acids but also raised the abundance of predominant flora, such as *Bacteroides*, *Firmicutes*, *Verrucomicrobia*, and *Proteobacteria*. These results implicate glucansucrase as an efficacious tool for the enzyme synthesis of oligoglucans. Furthermore, both polysaccharides with different degrees of polymerization may be beneficial in maintaining a healthy human gut.

## 1. Introduction

Extracellular polysaccharides (EPSs) from lactic acid bacteria are mucous or capsular polysaccharides that are produced and secreted extracellularly during the growth and metabolism of lactic acid bacteria [1]. *Sanfranciscensis*, *La. plantarum*, *La. reuteri*, *Streptococcus thermophilus*, *La. pentosus*, *Weissella cibaria*, *Leuconostoc mesenteroides*, and *La. helveticus* produce exoglycans. The components and functions of EPSs produced by different strains are highly diverse. EPSs were first used to improve the tolerance of lactic acid bacteria to their surrounding environment (water, temperature, pH, bile salt, etc.). With further knowledge gained from various studies, EPSs have been widely used in food processing and drug development, reflecting their broad-spectrum antibacterial activity, ability to inhibit the proliferation of cancer cells [2], antioxidant activity [3], contribution to cholesterol reduction [4], ability to improve food texture [5], ability to extend shelf life, and probiotic activity. Extracellular polysaccharides (BBE) isolated from *Bifidobacterium breve* are able to alleviate colitis by restoring the balance of the intestinal microbiota and increasing occludin, claudin-1 and ZO-1 expression, and mucin content [6]. It is likely that EPS-09 from *L. plantarum WLPL09* could adjust the composition of the intestinal microbiota (such as increase the abundance of phylum *Firmicutes* and lower the abundance of genus *Akkermansia*) and upregulate the transcription of apoptosis-related genes for inhibiting melanoma-bearing mice tumors [7]. The EPSs generated from *L. mesenteroides* XR1 consist of glucose and galactose, which contribute to a higher viscosity, elasticity, and water retention in fermented milk, which could be an alternative to xanthan gum in food additives [8]. Matsuzaki et al. [9] demonstrated the adjuvant activity of *L. mesenteroides* NTM048 EPS in mucosal vaccines in mice. In vitro fermentation experiments have revealed that α-glucan from *L. citreum* SK24.002 exhibits good probiotic properties in terms of both microbial and short-chain fatty acids (SCFAs) [10]. The EPSs above were obtained by the direct fermentation of different strains. Other common methods of preparation include hydrolysis, chemical synthesis, and enzymatic synthesis. The enzymatic method has been the main route for developing EPS in recent years owing to the advantages of the mild reaction conditions, controllability, and low environmental pollution.

Glucansucrase, also known as glucosyl transferase (GTF), belongs to the GH70 family of hydrolytic glycosidases according to the carbohydrate-active enzyme classification system, which is mainly produced by lactic acid bacteria. The enzyme synthesis of EPSs relies on the success of the acceptor reaction, comprising a glycosyl-donor (sucrose), a glycosyl-acceptor, and glucansucrase. The principle of the acceptor reaction is the transfer of some sucrose to other acceptor sugars, leading to oligosaccharides with varying degrees of glycosidic bonding, polymerization, and functional activities. Ispirli et al. [11] synthesized DP3-cellobiose-derived oligosaccharides with bifidogenic effects and prebiotic properties through the acceptor reaction of glucansucrase E81, cellobiose, and sucrose. *L. citreum* KM20-glucansucrase-catalyzed steviol glycoside transglycosylation products can serve as commercial sweeteners with improved stability [12]. Fisetin-4′-O-α-D-glucopyranoside (FST-G1) with glucansucrase from *L. mesenteroides* NRRL B-1299CB4 exhibited greater water solubility and anti-inflammatory effects [13]. There are various types of glucan acceptors; of these, maltose is the most effective for glucan and oligosaccharide synthesis [14]. As research continues, the market demand for oligosaccharides continues to expand. However, meeting this demand is hampered by output and costs.

Domestic and international efforts seek suitable strains of glucansucrase-producing bacteria to boost production and identify practical applications. Therefore, it is important to conduct in-depth research on the industrial production of glucan oligosaccharides and their functions. The objectives of this study were to explore the in vitro synthesis of glucan oligosaccharides by *L. mesenteroides* 6055 and to examine the structural and intestinal functional properties of glucans and oligoglucans.

## 2. Results

### 2.1. Properties of Glucansucrase from L. mesenteroides 6055

**Figure 1:**
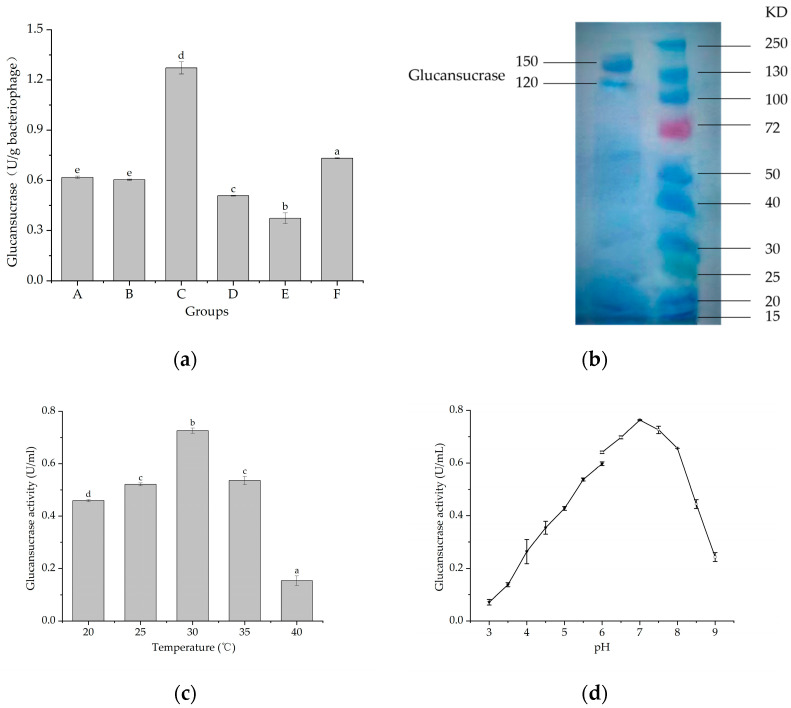
(**a**) Comparison of enzyme activities of glucansucrase from different parts (°C). Group A was *Leuconostoc mesenteroides* 6055 fermentation broth after 17 h incubation. Group B and group C were supernatant and bacterial precipitate of centrifuged group A, respectively. Group D and group E were supernatant and bacterial precipitate of group C after ultrasonication, respectively. Group F was group B after ultrafiltration and concentration. The lower-case letters in this figure represent the standard deviation. (**b**) SDS-PAGE of the intermediate fraction of glucansucrase. (**c**) Optimum reaction temperature *Leuconostoc mesenteroides* 6055 glucansucrase. The lower-case letters in this figure represent the standard deviation. (**d**) Optimum pH of *Leuconostoc mesenteroides* 6055 glucansucrase. The broken lines in this figure were due to the creation of different pH environments with an acetate buffer system (pH 3.0–6.0) and phosphate buffer system (pH 6.0–9.0) separately.

The glucansucrase activity in the samples obtained from different extraction and purification methods were significantly different, with activity being detected in all groups (A–F) (Figure 1a). The enzyme activity of group C was the highest (1.27 U/mL), which was followed by group F (0.73 U/g), group A (0.62 U/g), group B (0.60 U/mL), group D (0.51 U/mL), and group E (0.37 U/g). Ultrafiltrated glucansucrase was collected and identified by SDS-PAGE (Figure 1b). Two distinct protein bands with molecular weights of 150 and 120 kDa, respectively, were evident. Figure 1c,d show the effects of pH and temperature on the enzyme activity. The activity of the glucansucrase remained high (>0.4 U/mL) between 20 and 35 °C, showing a sharp decrease between 35 and 40 °C. The temperature of 30 °C was the optimal choice. Figure 1d indicates that the activity of the glucansucrase was positively correlated with pH values of 3.0–7.0. The enzyme activity changed significantly when the pH value was 8.0–9.0. The optimum pH was 7.0, and the enzyme was sensitive to alkaline conditions.

### 2.2. Analyses of Enzyme Synthesis

**Figure 2:**
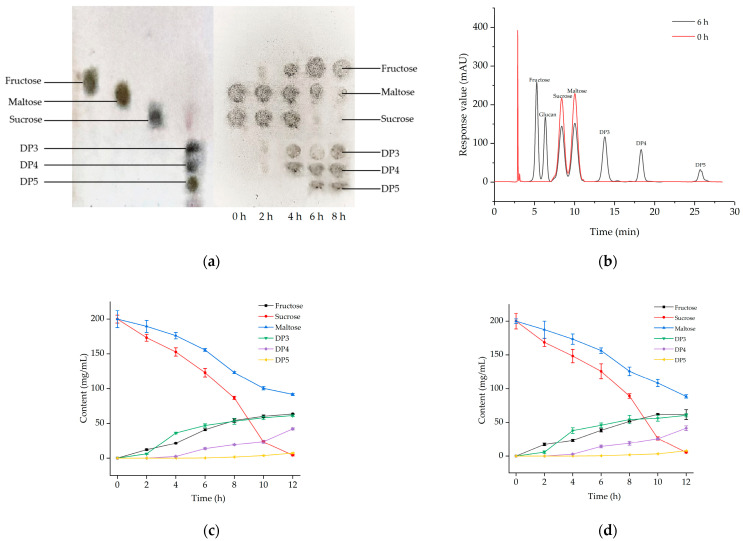
(**a**) Standard of different oligosaccharides (**left**) and the thin layer chromatogram of the synthetic products of sucrose and maltose catalyzed by the glucansucrase at different reaction times (**right**). (**b**) HPLC analysis of the products catalytically synthesized by the glucansucrase. (**c**) The change curves of main substance during fermentation of *Leuconostoc mesenteroides* 6055. (**d**) The change curves of main substances during the synthesis of oliglucans by glucansucrase.

Glucansucrase from *L. mesenteroides* 6055 catalyzed the synthesis of glucan-based oligosaccharides using sucrose as a donor. The composition of the products was then analyzed by TLC and HPLC (Figure 2).

#### 2.2.1. TLC Analysis of Oligosaccharide Products

As displayed in Figure 2a (left), the standard spots were fructose, maltose, sucrose, and commercial oligosaccharides (triosaccharides, tetrasaccharides, and pentasaccharides) in sequence. TLC results corresponding to the glucansucrase acceptor reaction at 0, 2, 4, 6, and 8 h are shown in Figure 2a (right). The substrate patches decreased in size, with the top fructose dot progressively becoming larger, and the three spots appearing in sequence becoming progressively larger as the reaction time increased. A comparison of the left and right graphs suggested that the first to be synthesized were triosaccharides (DP3), which were followed by tetrasaccharides (DP4) from a competitive glucosylation reaction using oligotriose and maltose as substrates, and finally pentasaccharides (DP5) inter-polymerized by tetrasaccharides. Additionally, the competitiveness of the glucosyl group of oligoglucan decreased with lengthening of the carbon chain, resulting in different yields in the order of DP3 > DP4 > DP5.

#### 2.2.2. HPLC Analysis of Oligosaccharide Products

As shown in Figure 2b, only the peaks of sucrose and maltose were visible before the reaction. After 6 h of the reaction, the sucrose and maltose peaks gradually decreased in area, while peaks for fructose, glucose, and three oligosaccharides appeared, with concentrations of 43.2 mg/mL, 28.6 mg/mL, 73.2 mg/mL, 36.5 mg/mL, and 9.4 mg/mL, respectively. The HPLC retention times indicated that product 1 was DP3 (13.28 min), product 2 was DP4 (21.45 min), and product 3 was DP5 (35.13 min), which agreed with the TLC results.

#### 2.2.3. Comparison of Microbial Fermentation and Enzyme Synthesis

The changes in the content of each substance in the enzymatic synthesis and direct strain fermentation were compared (Figure 2c,d). The trends of changes of each substance in the two synthesis methods were identical, but the yields were slightly different. The oligoglucan production from enzymatic synthesis was 141.99 mg/mL; however, that of *L. mesenteroides* 6055 fermentation was 103.12 mg/mL. These results indicated that enzymatic synthesis was more efficient than strain fermentation. Therefore, glucansucrase enzymatic synthesis could be applied to industrial production to improve oligosaccharide output.

### 2.3. Analysis of Physicochemical Properties of Glucan and Oligoglucan

#### 2.3.1. SEM, Zeta Potential, and Viscosity Analysis

**Figure 3:**
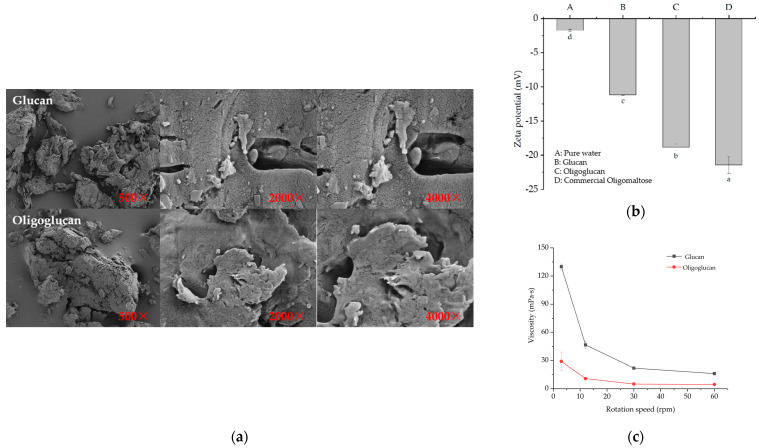
(**a**) The glucan and the oligoliglucan at a magnification of 500×, 2000× and 4000× under SEM pictures. (**b**) Zeta potential analysis of the glucan and the oligoglucan solution. A was the result of pure water, and B for the glucan, C for the oligoglucan, D for the commercial oligomaltose. The lower-case letters in this figure represent the standard deviation. (**c**) Viscosity curves of the glucan and the oligoglucan solutions under different shear forces.

SEM revealed the different morphological characteristics of the two polysaccharides (Figure 3a). The glucan was loose in structure, dispersed, and fibrous in form. Polysaccharides with these characteristics can form a consistent matrix of hydrated polymers to improve the physical and chemical properties of products, such as viscosity, solubility, and holding capacity, to promote their wide application in food, cosmetics, and other fields [15,16]. The oligoglucan had a compact structure and was dispersed in lumps. These characteristics would permit use in the preparation of malleable membrane materials with potential biomedical applications. The zeta potential values for glucan and oligoglucan are depicted in Figure 3b. The zeta potential values of the different kinds of polysaccharides were negative, indicating that the experimental polysaccharide solutions were all negatively charged. The potential values were ranked in order of pure water < the oliglucan (−18.83 ± 0.49 mV) < the glucan (−11.18 ± 0.15 mV) < the commercial oligomaltose (−21.45 ± 0.23 mV). Thus, the stability of the oligoglucan was greater than that of the glucan but worse than that of the commercial oligomaltose. The viscosity of the glucan and the oligoglucan sample solutions at 24 °C was determined using a rotary viscometer (Figure 3c). When the shear rate was increased from 3 to 60 rpm, the viscosity of the glucan solution decreased from 130 ± 2.82 mPa·s to 16.15 ± 0.77 mPa·s, while the viscosity of the oligoglucan solution declined from 29 ± 9.89 mPa·s to 4.55 ± 0.77 mPa·s. Meanwhile, at the shear rate of 3 rpm, the viscosity value of the glucan solution was significantly higher than that of the oligoglucan solution. In addition, the viscosities of the two polysaccharide solutions gradually dropped and tended to be the same as the shear rate increased.

#### 2.3.2. Thermodynamic Analysis

**Figure 4:**
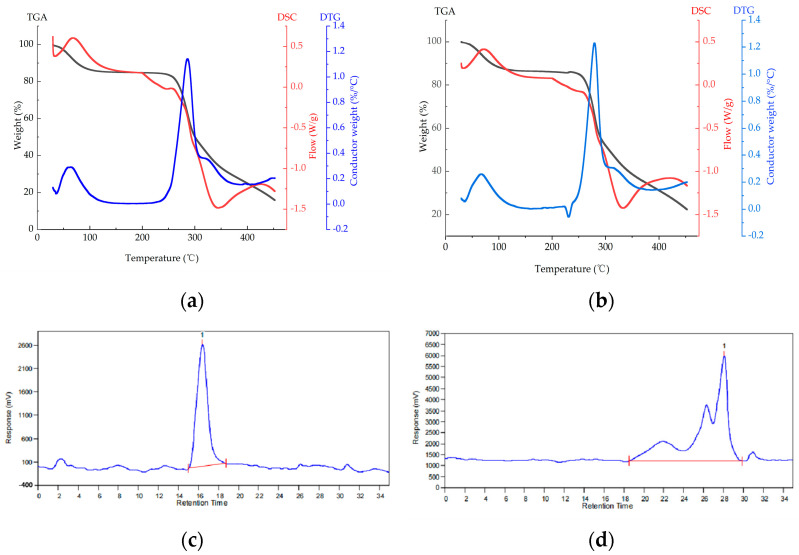
(**a**,**b**) Thermal property analysis curves of the glucan (**a**) and the oligoglucan (**b**). The black curves were TGA curves, the red curves were DSC curves, and the blue curves were DTG curves. (**c**,**d**) HPSEC chromatogram of the glucan (**c**) and the oligoglucan (**d**). The number 1 means the highest peak of the glucan and the oligoglucan, respectively.

The thermodynamic analysis curves for the glucan and the oligoglucan are presented in Figure 4a and 4b, respectively. The degradation process was divided into three stages. In the first stage, from the initial temperature of 29.57 to 99.7 °C, approximately 15% of the mass loss occurred in the two groups. This could be attributed to the fact that the polysaccharide molecules contained a large number of carboxyl groups bounded to water molecules. As the temperature increased, this part of the water would gradually be lost. In the second stage, when the temperature continued to increase to 450 °C, the maximum mass loss of polysaccharides was approximately 65%, which was mainly due to the depolymerization of the polysaccharide samples in the interval, which showed a large step in the curves. Finally, with the increasing temperature, the complex molecular structure of the polysaccharide, such as sulfuric acid groups, caused the samples to reach constant weight.

A sharp peak on the derivative thermogravimetry curve was evident (Figure 4a,b). The peak was the highest point on this curve and the result of the most dramatic change in sample mass. The maximum rate of weight loss for the glucan and the oligoglucan occurred at 286.54 °C and 280.24 °C, respectively [17].

The DSC curves revealed three melt heat absorption peaks for the glucan (33.26 °C, 278.93 °C, and 343.44 °C) and the oligoglucan (33.51 °C, 262.46 °C, and 332.98 °C) with little difference between the two polysaccharides. The first heat absorption peaks appeared at 33 °C. Between 33 and 99.7 °C, the water in the samples evaporated and absorbed heat. The second peaks at 270 °C were melt heat absorption peaks, and no weight loss occurred. The third heat absorption peaks were at 340 °C, after which the two polysaccharides melted completely into crystals and did not change significantly with increasing temperature.

The collective findings indicated that the glucan and oligoglucan prepared in this study have broad prospects for applications in the food thermal processing industry and could be used to improve the heat resistance of foods, such as bread.

#### 2.3.3. Molecular Weight

The molecular weights of the glucan and the oligoglucan samples were determined using high-performance gel permeation chromatography. The glucan displayed a single symmetric peak with a polydispersion index of 1.32432 (Figure 4c,d and, Table 1). The closer the polydispersion index is to 1, the more homogeneous the material is. The molecular weight of the glucan exceeded 10 kDa, indicating that the polysaccharide fraction was homogeneous. The number average molecular weight of the glucan was 8.4 × 10^5^ Da, while the weight average molecular weight was 1.1 × 10^6^ Da. In general, glucans produced by bacteria have molecular weights between 10^5^ and 10^9^ Da. There were three elution peaks corresponding to the three oligosaccharides, with a number average molecular weight of 725 Da and a weight average molecular weight of 3342 Da, both of which were much lower than those of the glucan (Figure 3g). The molecular weight of the polysaccharide depends on the strain and substrate components that include sucrose content, reaction conditions, and other factors. The higher the sucrose content of the substrate, the greater the relative molecular weight of the polysaccharide produced [18].

#### 2.3.4. XRD Analysis

**Figure 5:**
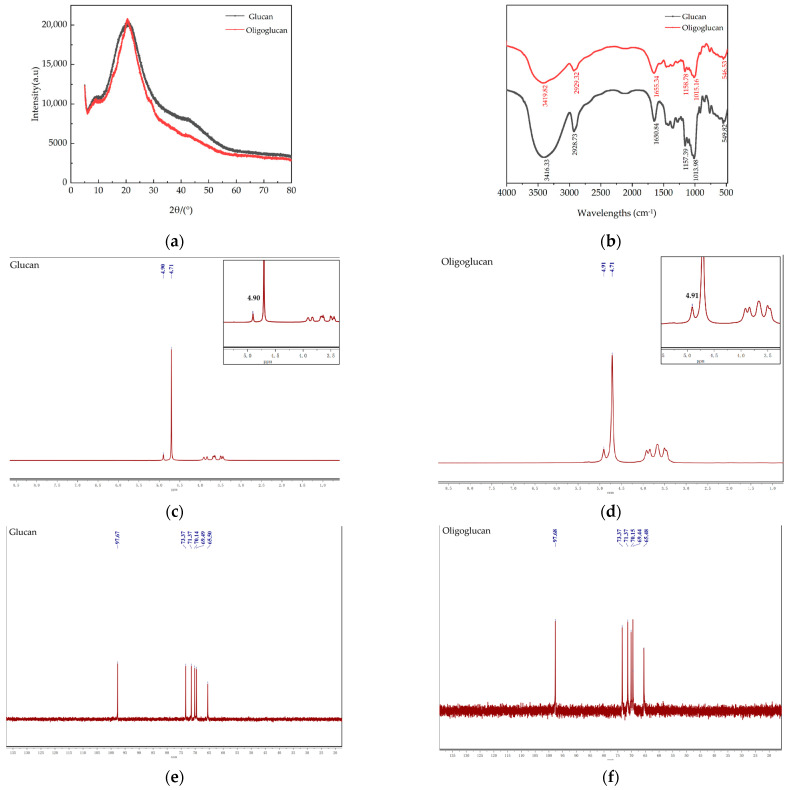
(**a**) X-ray diffraction patterns of the glucan and the oligoglucan. (**b**) FT-IR spectrogram of the glucan and the oligoglucan. (**c**,**d**) ^1^H-HMR spectra of the glucan and the oligoglucan. (**e**,**f**) ^13^C-HMR spectra of the glucan and the oligoglucan.

The XRD pattern of the glucan and the oligoglucan between 5° and 80° indicated that they were semi-crystalline or amorphous substances (Figure 5a). The strong diffraction peaks appeared when 2θ was 20°, and the wide diffraction peaks with a weak signal appeared near 40°, indicating that the overall crystallinity of polysaccharides was low, and most polysaccharides existed in an amorphous form.

#### 2.3.5. Fourier Transform-InfraRed (FT-IR) Analysis

The FT-IR spectra of the glucan and the oligoglucan are presented in Figure 5b. The wide and strong absorption peak at 3447.69 cm^−1^, which was the characteristic absorption peak of polysaccharides [19], reflected O-H stretching vibration and the peak at 2928.31 cm^−1^, which was due to the stretching vibration of the alkyl C-H bond. The absorption bonds at 1652.03 and 1157.28 cm^−1^ could be attributed to the asymmetric stretching vibration of carboxyl C=O and the α-glucoside bonds in the main chain of the glucan and the oligoglucan, respectively. In addition, the absorption peak at 1014.84 cm^−1^ was assigned to stretching vibrations of the pyran ring in glucose residues, inferring that the monosaccharide residues in samples existed as an α-configuration pyran ring. With the increase in chain length, the characteristic peak value increased gradually.

#### 2.3.6. Nuclear Magnetic Resonance (NMR) Analysis

The ^1^H NMR spectra of glucan and oligoglucan are shown in Figure 5c,d. The 4.5–5.5 ppm range corresponds to the heterohead region of the glucan and the oligoglucan, and 3.1–4.5 ppm corresponded to the cyclic proton region of C2–C6 [20]. ^1^H NMR revealed that the peak spectra of the two polysaccharides did not differ much, with a strong signal peak at 4.90 ppm in the heterotopic region for both glucan and oligoglucan, which is consistent with the absorption peaks appearing at 1014.84 cm^−1^ in the IR spectrum, representing an α-1,6 glycosidic bond. The strong peak at 4.71 ppm was due to D_2_O, suggesting that the glucan and the oligoglucan were mainly polymers linked by α-1,6 glycosidic bonds. 

Figure 5e,f depict the ^13^C NMR spectra of the glucan and the oligoglucan. For the spectra of glucan produced by *L. mesenteroides* TDS2-19, the peaks at 97.67, 73.37, 71.37, 69.44, and 65.50 ppm corresponded to C-3, C-2, C-5, C-4, and C-6 substituted glucose residues, respectively [21]. Based on these findings, the C-3, C-2, C-5, C-4, and C-6 positions of the glucan and the oligoglucan in this study could be deduced. In the ^13^C NMR spectrum, chemical shifts in the range of 95–110 ppm indicated the heterohead carbon region, and those in the range of C 50–85 ppm denoted the cyclic carbon region. The heterohead carbon of the glucan was evident at C 97.67/73.37/71.37/70.14/69.49/65.50 and that of oligoglucan appeared at 97.68/73.37/71.37/70.15/69.44/65.48. The downward shift of the C6 carbon signal of the glucose unit at 65.48 ppm for the glucan and the oligoglucan indicated that the glucose united in the polymer backbone was linked by α-1,6 glycosidic bonds. No additional peaks were observed in the range of 75–85 ppm, indicating the absence of other branched chains. The allosteric carbon signal at 97.67 ppm for the glucan and 97.68 ppm for the oligoglucan indicated that both of them were ligated by α-glycosidic bonds to glucose residues. Collectively, these findings demonstrated that both glucan and oligoglucan were linear structures with α-1,6 glycosidic bonds linking the main chains, which was accompanied by the branching of glucose residues linked by α-type glycosidic bonds. The results are consistent with the FT-IR scan data.

### 2.4. In Vitro Simulation of Intestinal Probiotic Function

#### 2.4.1. Changes of pH and Strain Density during Fermentation

**Figure 6:**
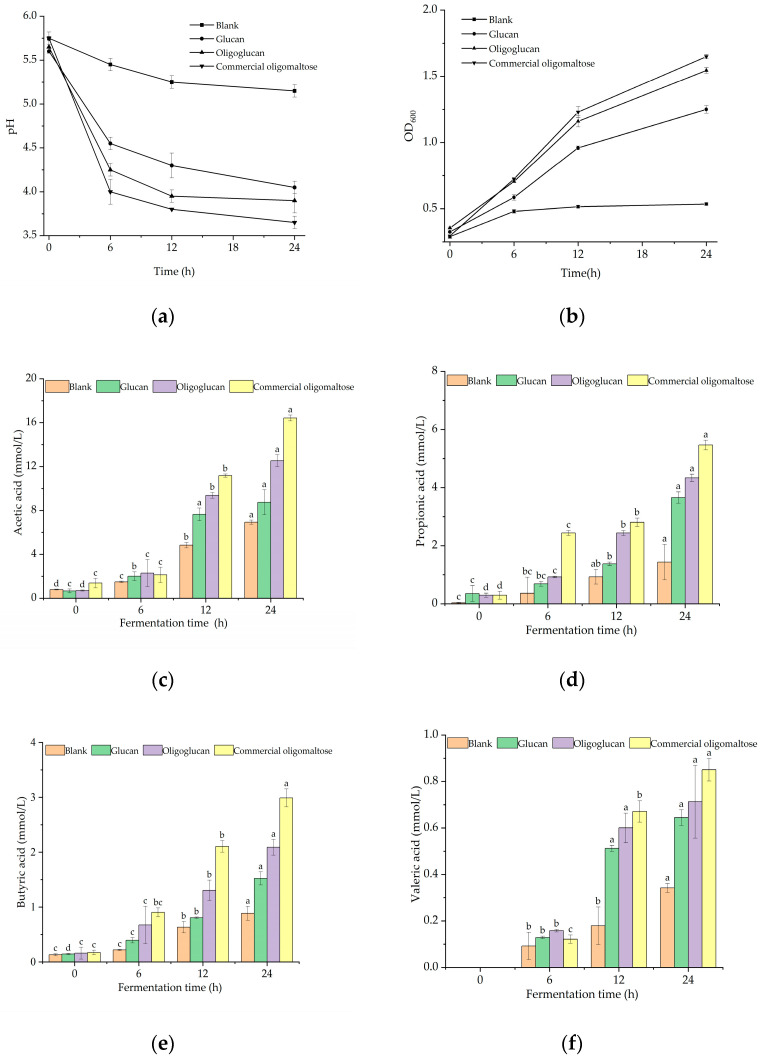

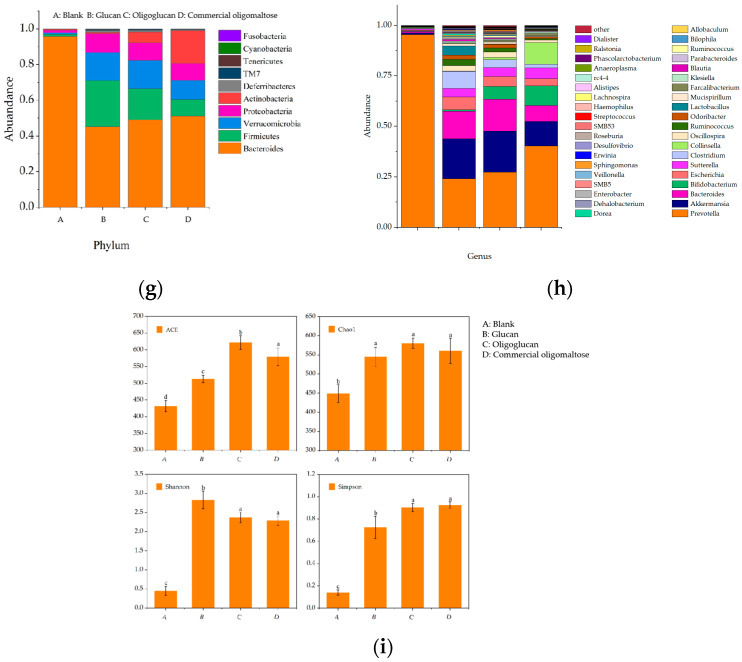
(**a**,**b**) Changes of pH and OD_600_ in different carbon source groups during simulated fermentation in vitro. (**c**–**f**) Synthesis of acetic acid, propionic acid, butyric acid and valeric acid by simulated anaerobic fermentation of intestinal bacteria with different carbon source groups in vitro. The lower-case letters in Figure 6c–f represent the standard deviation. In (**g**–**i**), A represents the blank group, B represents the glucan group, C represents the oligoglucan group, and D represents commercial oligomaltose. (**g**,**h**) Effects of different carbon sources on intestinal microflora (phylum and genus). (**i**) Abundance and diversity of intestinal microflora after fermentation with different carbon sources. The lower-case letters in this figure represent the standard deviation.

The pH of the fermentation broth with the glucan, the oligoglucan, and the commercial oligomaltose as carbon sources dramatically decreased during the in vitro simulated fermentation process except for the blank group (Figure 6a,b). The pH of the fermentation broth declined most rapidly from 0 to 6 h, falling from an initial pH of 5.7 to 4.0, 3.91 and 3.65 in the glucan, oligoglucan, and commercial oligomaltose groups, respectively, and reaching equilibrium after 24 h. A drop in intestinal pH reduced the pathogenic microbial attack and accumulation of toxic metabolites. According to the overall trend of OD_600_, the fermentation broth in the three different groups rose consistently during 24 h, from 0.3 to 1.25, 1.55, and 1.65 in the glucan, oligoglucan, and commercial oligomaltose groups, respectively. The quickest increase from 0 to 6 h suggested that the intestinal flora proliferated rapidly during this period.

The glucan, the oligoglucan, and the commercial oligomaltoses all exhibited positive effects on gut microbiota proliferation. Overall, commercial oligomaltose > oligoglucan > glucan. The findings differed from the previous pattern of oligoglucan > glucan > commercial oligomaltose. Moreover, the proliferative values of the two oligosaccharides were approximately 1.5–1.7, which were significantly higher than that of glucans (1.25). Differences in the structures of the three could contribute to the above results. The speed of microbial proliferation was limited by the degree of oligosaccharide polymerization. A low degree of polymerization leads to a high microbial utilization ratio and proliferation rate, which was also the reason for the slow growth of glucan groups in the early stage of fermentation [22].

#### 2.4.2. Production of SCFAs during Fermentation

The low pH of the microbial environment was the result of the accumulation of SCFA. Under the influence of fermentation substrates, microbial species, and other factors, SCFAs from intestinal microorganisms vary in type and quantity, and their physiological functions are also different [23]. The concentrations of SCFAs from the glucan, the oligoglucan, and the commercial oligomaltose during simulated intestinal fermentation in vitro are displayed in Figure 6c–f. The glucan group produced 8.74 mmol/L of acetic acid, 3.65 mmol/L of propionic acid, 1.52 mmol/L of butyric acid, and 0.61 mmol/L of valeric acid. Acetic acid accounted for the highest proportion of 60.19%. In the oligoglucan group, the yields of acetic, propionic, butyric, and valeric acids were 12.54, 4.33, 2.09, and 0.71 mmol/L, respectively. Acetic acid was the most abundant at 63.75%. The control commercial oligomaltose group produced 16.43 mmol/L acetic acid, 5.46 mmol/L propionic acid, 3.66 mmol/L butyric acid, and 0.85 mmol/L valeric acid. Acetic acid again displayed the maximum percentage yield at 62.23%. The specific weight of the composition of SCFAs in different groups was acetic acid > propionic acid > valeric acid. The output of SCFAs in the glucan, oligoglucan, and commercial oligomaltose groups was higher than that in the blank control group.

#### 2.4.3. Changes in Intestinal Microbial Flora Growth

To assess the overall structural changes in gut microbial composition during in vitro simulated fermentation, the relative abundance of major bacterial groups was assessed at the phylum (Figure 6g) and genus (Figure 6h) levels. As shown in Figure 6g, at the phylum level, the four most abundant groups in the glucan- and oligoglucan-treated and commercial oligomaltose control groups were *Bacteroides*, *Firmicutes*, *Verrucomicrobia*, and *Proteobacteria*. At the genus level (Figure 6h), there were significant differences in the gut microbiota before and after treatment. The abundance of *Akkermansia*, *Bacteroides*, and *Bifidobacterium* demonstrated increasing trends after treatment with polysaccharides. Figure 6i summarizes the results of the abundance and diversity of intestinal flora after polysaccharide fermentation with different indices. The values of the ACE, Chao1, Shannon, and Simpson’s indices were higher in the glucan, oligoglucan, and commercial oligomaltose groups than in the blank control group.

## 3. Discussion

### 3.1. Properties of Glucansucrase from L. mesenteroides 6055

In the comparison of groups A–F, group F was markedly lower than group C. However, since group C was the bacterial precipitate of fermentation, the enzyme yield was too low to be purified, which was not conducive to the subsequent experiments. Therefore, the ultrafiltration supernatant (group F) was used to purify the enzyme solution. In addition, the fermentation supernatants of groups B and C had corresponding enzyme activities, demonstrating that glucansucrase could be an extracellular and cell wall enzyme. The molecular weights of glucansurcrase have two distinct protein bands, which were comparable with glucansucrase from *Leuconostoc mesenteroides* NRRL B-640 and *Weissella cibaria* JAG8. They were reported as 180 and 177 kDa respectively [24]. Song et al. also observed that *Leuconostoc citreum* SK24.002 glucansucrase had a molecular weight at 186 kDa. These values were slightly higher than the results of this study, which may relate to different strains. The optimum temperature at 30–35 °C was similar with *Lactobacillus reuteri* SK24.003 glucansucrase at 30–35 °C [25] and *Leuconostoc lactis* EG001 at 25–35 °C [26]. And the optimum pH was neutral and sensitive to alkaline environments, which was because the acidity or alkalinity of the solution not only affected the structural integrity and stability of enzyme proteins but also influenced the dissociation state between them and the corresponding substrates; therefore, only in the optimal pH interval of enzyme protein molecules can their catalytic activity be expressed to the maximum extent. Most strains were able to maintain the cytoplasm in a neutral environment, allowing metabolic and various enzymatic reactions to proceed normally [27].

### 3.2. Analyses of Enzyme Synthesis

By TLC and HPLC (Figure 2a,b), the compositions of oligoglucan products were evidenced including four oligoglucans. Similarly, the oligoglucans generated by the dehydration condensation of glucose in concentrated sulfuric acid were investigated by Zeng et al. [28] using TLC, in which oligoglucans with polymerization degrees between 1 and 4 were well separated. And in 2017, Miao et al. studied the glucansucrase produced by *Lactobacillus reuteri* SK24.003 and found that the products were maltotriose, maltotetrasaccharide, and maltopentasaccharide linked by α-1,4-/α-1,6-glycosidic bonds when maltose was used as the acceptor [25].

### 3.3. Analysis of Physicochemical Properties of Glucan and Oligoglucan

In structure studies (Figure 3a–c), SEM differences between the two polysaccharides could be related to their different structural characteristics, such as polymerization degree and molecular weight. For example, Goyal et al. [29] reported that the microscopic structure of *L. mesenteroides* NRRL B-1149 purified polysaccharide was fibrous. Yang et al. [30] found that the polysaccharide of *L. citreum* NM105 has a flaky, highly branched structure with a smooth and shiny surface. The zeta potential and solution stability have a correlation: the higher the absolute value of the zeta potential, the greater the repulsive force between the charged particles in the solution, thus promoting the stability of the solution; and the lower the absolute value of the zeta potential of the solution, the more the attraction between the particles overcomes the repulsive force, the more the molecules would be aggregated, the more unstable the solution. In general, when the absolute value of the zeta potential of a solution is more than 20, the physical properties of the solution are extremely stable. And zeta potential values could be related to the degree of polymerization of polysaccharides, linkage mode of glycosidic bonds, the extraction method of polysaccharides and so on. Cheng et al. [31] found that the change trend of zeta potential of polysaccharide solutions obtained by spray drying and ethanol precipitation drying was markedly different when the pH was >3.5. Solution viscosity is normally associated with intermolecular hydrogen bonding forces, and changes in viscosity have the ability to indirectly reflect changes in the structure of the sample. When the shear rate was increased from 3 to 60 rpm, the viscosity of the glucan solution decreased dramatically, which may be attributed to the fact that the structure of the glucan was disrupted during the process of shearing, showing good pseudoplastic rheological properties. Furthermore, the viscosity also depends on molecular weight, temperature, and pH. For example, EPS from *Lactobacillus plantarum* HM47 showed higher viscosity at lower temperature and acidic pH. In XRD analysis (Figure 5a), the overall crystallinity of polysaccharide was low, and most polysaccharides existed in an amorphous form. Similarly, the water-soluble polysaccharide obtained from potato peel showed a strong diffraction peak when 2θ approached 16.96° [32]. In addition, the polysaccharide isolated from Hohenbuehelia serotina was found to be a semi-crystalline or amorphous substance with a circular convex crystal reflection at 22.5° after selenium modification and before treatment [33]. Collectively, these findings confirmed that polysaccharides coexist in many amorphous forms and a small number of crystal states.

### 3.4. In Vitro Simulation of Intestinal Probiotic Function

In simulated fermentation, as shown in Figure 6c–f, the glucan, oligoglucan, and commercial oligomaltose groups promoted fermentation to produce more acetic acid, propionic acid, butyric acid and valeric acid. Among these results, acetic acid had the highest production, which was similar to that of r-EPS 1 from *L. delbrueckii* ssp. *Bulgaricus* SRFM-1 [34]. Intestinal flora can degrade undigested polysaccharides for conversion into SCFAs, such as acetic acid, propionic acid, and butyric acid, as catalyzed by carbohydrase [35]. A variety of microorganisms in the intestinal flora can produce and accumulate acetic acid, such as *Bifidobacterium*, *Bacteroides*, *Eubacterium*, etc., which are the main substances for microorganisms to provide energy for the host. Propionic acid, a major metabolite of *Bacteroides*, inhibits the activity of 3-hydroxy-3-methylglutaryl coenzyme A reductase and prevents cholesterol synthesis [36]. Butyric acid is the signal product of *Firmicutes* fermentation and is absorbed and utilized by epithelial cells. Butyrate interferes with the proliferation and differentiation of tumor cells and plays an important preventive role in colitis and colon cancer [37]. Oligoglucans and glucans obtained from maltose have good intestinal biocompatibility, which can effectively promote the proliferation of beneficial intestinal bacteria, control intestinal pH at an acidic level, and reduce risks of intestinal diseases.

And in Figure 6g, among the four most abundant microbiota at the phylum level, *Bacteroides* has been extensively investigated for its regulatory effects on the host. In addition to contributing to the catabolism of complex polysaccharides, proteins, and lipids in meeting normal growth and metabolic nutrient energy requirements, along with promoting the growth of other bacteria, *Bacteroides* is the typical SCFA-producing bacterium in the gastrointestinal tract. *Bacteroides* is beneficial for maintaining intestinal homeostasis, enhancing intestinal health, and reducing low-grade inflammation [38]. Compared with the blank group, *Firmicutes* and *Proteobacteria* were elevated following the glucan and oligoglucan intervention treatments. Both phyla have been reported to be the major bacteria in the human gut. Moreover, obesity can be inhibited by regulating the structure of the gut microbiota [39]. In addition, different polysaccharides had varying effects on the intestinal flora, with glucan being more effective in *Firmicutes* proliferation, oligoglucan being more productive for *Proteobacteria* growth, and commercial oligomaltose being more efficient in promoting *Bacteroides*, which may be related to the variance in the structure and properties of polysaccharides. As for the genus level (Figure 6h), glucan and oligoglucan primarily enhanced the abundance of *Akkermansia* and *Bacteroides*. *Akkermansia* is the representative bacteria of the human intestinal species of *Verrucomicrobia* phylum, which could exert metabolic protection by modifying the integrity of the intestinal epithelium and mucus layer as well as by regulating T cells to perform anti-inflammatory effects [40]. Commercial oligomaltose significantly increased the abundance of *Bifidobacterium*. *Bifidobacterium* is a beneficial intestinal bacterium that not only maintains the balance of normal intestinal flora, avoiding constipation, diarrhea, etc., but also, in a similar way as *Bacteroides*, synthesizes SCFAs in the host intestine, the intestinal species of *Verrucomicrobia* phylum, which could exert metabolic protection by modifying improving immunity, lowering blood cholesterol levels, and preventing hypertension [41].

The findings in Figure 6i indicated that the addition of the glucan and oligoglucan led to an enhancement in the abundance and diversity of the intestinal flora. The highest ACE and Chao1 index values were achieved in the oligoglucan group, the Shannon index was highest in the glucan group, and the Simpson index was highest in the commercial oligomaltose group. The ACE, Chao1, Shannon, and Simpson indices measure α-diversity. In addition, the ACE and Chao1 indices reflect the richness of microbial populations. In these foregoing indices, higher values indicated progressively richer microbial populations. Shannon and Simpson indices are indicators of the diversity of microbial populations in samples; proportionally, the greater the value, the more diverse the microbial population [42]. Therefore, to achieve microbial proliferation, the oligoglucan was principally introduced by increasing the abundance of microbial populations in the gut, while the glucan and the commercial oligomaltase were principally introduced by raising the diversity of microbial populations in the gut. The better the richness and diversity of microbial populations in the gut, the higher the immunity and resistance of the individual. These results suggested that the glucan and the oligoglucan have prebiotic characteristics and play positive roles in maintaining intestinal microbial richness and diversity [43].

## 4. Materials and Methods

### 4.1. Preparation and Cultivation of Bacterial Strains

*L. mesenteroides* CICC6055 obtained from the China Industrial Microorganism Preservation Center (CICC) was activated in glucansucrase medium for 2–3 generations and then inoculated in sterilized glucansucrase medium at a 2% ratio and incubated at 28 °C for 17 h. The glucansucrase medium consisted of 20 g/L sucrose, 5 g/L yeast extract, 5 g/L peptone, 0.2 g/L MgSO_4_, 3.0 g/L KHPO_4_, 0.05 g/L CaCl_2_, and 5 mL/L Tween-80 and then was sterilized at 121 °C for 15 min.

### 4.2. Extraction of Exopolysaccharides by L. mesenteroides 6055

#### 4.2.1. Glucans from Strains Fermentation

Strain CICC6055 was inoculated (3% *v*/*v*) into modified De Man, Rogosa and Sharpe liquid medium and incubated at 28 °C for 48 h (Vertical pressure steam sterilizer: YXQ-50SII, Shanghai Boxun Medical Biological Instrument Corp, Shanghai, China). The culture was centrifuged at 8000 rpm, 4 °C for 5 min. Then, the fermentation supernatant was collected and stored in a high-speed frozen centrifuge (GL-20G-II, Shanghai Anting Scientific Instrument Factory, Shanghai, China). Trichloroacetic acid (TCA) was added to the supernatant until the concentration of TCA in the fermentation supernatant reached 5% (*m*/*v*) for removing protein. After the precipitation was no longer increasing, the solution was centrifuged at 8000 rpm, 4 °C for 20 min. And for the supernatant without protein, its pH was adjusted to neutral and was first dialyzed in distilled water at 10 kDa for 72 h and then in anhydrous ethanol overnight. At last, the precipitate in a dialysis bag was concentrated and lyophilized as a glucan sample.

#### 4.2.2. Analyses of Properties of Glucansucrase

##### Extraction, Purification, and Enzyme Activity Assay

Enzyme activities were measured in A–F groups to determine the best extraction method. *Leuconostoc mesenteroides* 6055 fermentation broth after 17 h incubation is group A. The fermentation broth of group A was centrifuged at 6000 r/min, 4 °C for 8 min, and the resulting supernatant and bacterial precipitate were group B and group C, respectively. Then, 20 mL of fermentation supernatant was centrifuged in a 10 KDa ultrafiltration tube at 8000 r/min, 4 °C for 20 min, and the fermentation supernatant was replenished so that 50 mL of supernatant was concentrated to 5 mL to obtain group F. The precipitate of group C was resuspended in phosphate buffer solution (pH 7, 20 mmol/L), centrifuged twice at 8000 r/min, 4 °C for 20 min each time, and then the phosphate buffer solution was added again. Afterwards, it was ultrasonically broken for 20 min (power 200 W, ultrasonication on for 1 s, off for 2 s), and finally, the ultrasonication solution was centrifuged at 8000 r/min, 4 °C for 20 min (Ultrasonic cell crushing instrument: Q700, Qsonica LLC, Newtown, CT, USA). The obtained supernatant and precipitate were groups D and E, separately.

In this study, enzyme activity was defined as the amount of enzyme (U) required to generate 1 µmol fructose from sucrose per minute. Fructose content was determined using the dinitrosalicylic acid method. The enzyme reaction system consisted of 100 mg/mL sucrose, 1 mmol/L CaCl_2_, 20 mmol/L phosphate buffer, and enzyme solution. The activities of the glucansucrase at different temperatures (20, 25, 30, 35, and 40 °C) and different pHs (3.0–9.0) were evaluated to optimize the enzymatic reaction conditions (Digital display thermostatic double-well water bath, BHS-2, Ningbo Yinzhou Qunan Experimental Instrument Co, Ningbo, China; pH meter, HI98103, Hanna Instruments, Shanghai, China).

##### Sodium Dodecyl Sulfate Polyacrylamide Gel Electrophoresis (SDS-PAGE)

First, 100 µL of sample buffer and 100 µL of enzyme concentrate to be tested were mixed in a 2 mL EP tube and sampled after 2 h in a 37 °C water bath. High-molecular-weight standard proteins were selected as the control, and electrophoresis was started at 30 mA current. At the end of the process, the film was removed and decolorized after staining with Coomassie brilliant blue (CBB), and the molecular weight size of glucansucrase was estimated with reference to the standard protein.

##### Oligoglucans from Enzyme Synthesis

A 10 mL glucansucrase reaction system was fabricated by adding 10% (*w*/*v*) sucrose, 10% (*w*/*v*) maltose, and the appropriate amount of enzyme solution. The reaction was carried out at the optimum temperature and pH and terminated by boiling in a water bath for 10 min every 2 h. The samples were obtained after centrifugation and ultrafiltration. Thin-layer chromatography (TLC) and high-performance liquid chromatography (HPLC) analyses were used to investigate the reaction process.

##### Thin-Layer Chromatography (TLC) Analysis

First, 0.2 µL of 10-fold diluted reaction solution was spotted on a silica gel plate and blown dry rapidly, and then it was placed into the development tank for development, and the reaction was stopped when the spreading distance was 1 cm from the upper end of the silica gel plate where the developer was a mixture of n-propanol, n-butanol, and deionized water (v:v:v = 5:3:4). After the development was completed at room temperature, the plate was air-dried in a cool place, then sprayed with a color agent (sulfuric acid: methanol v:v = 1:4), and finally dried in an oven at 110 °C for 10 min. The color development of the silica gel plates was observed.

##### High-Performance Liquid Chromatography (HPLC)

First, 10 mg/L glucose, fructose, sucrose, maltose, and panose standard solutions were prepared in deionized water, and their retention times were measured after membrane filtration. The oligoglucan content in reaction solution were ascertained by the normalization method using panose as the standard. HPLC was performed using an NH_2_ column (4.6 × 150 mm), differential refraction detector (refractive index detection, RID), mobile phase comprised of acetonitrile:water (80:20 v:v), flow rate of 1.0 mL/min, column temperature of 35 °C, and sample volume of 10 μL (High-Performance Liquid Chromatograph, LC-20AT, Shimadzu Corporation, Kyoto, Japan).

In a separate experiment, changes in each product during saccharogenesis (2, 4, 6, 8, 10, and 12 h) by fermentation and enzyme synthesis were determined and compared to verify the feasibility of biosynthesis.

### 4.3. Analysis of Physicochemical Properties of Glucan and Oligoglucan

#### 4.3.1. Scanning Electron Microscopy (SEM)

SEM examined the macroscopic surface morphology of the extracted and purified glucan and oligoglucan (Field Emission Scanning Electron Microscope, ZEISS Gemini 300, Carl Zeiss AG, Oberkochen, Germany). The freeze-dried polysaccharide powders were each adhered to a sample grid and coated with gold using an ion sputtering instrument. Different magnifications (500, 2000, and 4000×) images of the glucan and oligoglucan were obtained at an accelerating voltage of (10 kV).

#### 4.3.2. Zeta Potential

Zeta potentials of the glucan and the oligoglucan samples were determined using a Marvin nanoparticle and zeta potential analyzer (Zetasizer Nano ZS90, Malvern Instrument, Malvern, UK). The solubility of different polysaccharides was analyzed with pure water and commercial oligomaltose as controls. The operating mode of the instrument was set to automatic scanning. Each sample was scanned five times.

#### 4.3.3. Apparent Viscosity

A polysaccharide solution (10 mg/mL) was prepared by the dissolving freeze-dried glucan and oligoglucan (3, 12, 30, and 60 rpm) in a rotating viscometer (NDJ-79B, Shanghai Changji Geological Instrument Co., Ltd., Shanghai, China).

#### 4.3.4. Thermodynamic Properties

The thermodynamic properties of the polysaccharide samples were analyzed by thermogravimetry and differential scanning calorimetry (Synchronous thermal analyzer, TA Q600, Thermo Fisher Scientific, Waltham, MA, USA; DSC 8000, Perkin Elmer Instruments Co., Ltd., Hopkinton, MA, USA). Ten milligrams of each sample was placed in an Al2O3 crucible with an argon flow rate of 50 mL/min, temperature from 37 to 450 °C at a rate of 10 °C/min. Finally, the relationship curves between polysaccharide weight, melting point, and temperature were recorded and depicted.

#### 4.3.5. Molecular Weight Distribution

The molecular weight distributions of the samples were determined by HPLC. Glucan standards (10 mg/mL each) of different molecular weights were prepared. Their retention times were plotted as the logarithm of the standard molecular weight using LC-20AT (Shimadzu Corporation, Kyoto, Japan). Subsequently, 1 mg/mL glucan and oligoglucan solutions were prepared. The molecular weight was based on retention time calculations.

#### 4.3.6. Crystalline Configuration

After full grinding to eliminate particles, the sample powders were laid flat in the sample pool and measured using an X-ray diffractometer (Rigaku Ultima IV, B Rigaku Corporation, Tokyo, Japan). The rated output power was set to 3 KW with a rate of 2°/min.

#### 4.3.7. Fourier Transform-Infrared Spectroscopy (FT-IR)

Polysaccharide samples and potassium bromide were fully ground in a dry environment and pressed into a transparent sheet. The IR spectra of the samples were collected. The resolution ranged from 400/600 to 4000 cm^−1^ with 32 sweeps at a setting of 4 cm^−1^ (Fourier transform-infrared spectroscopy, Nicolet iS20, Thermo Fisher Scientific, Waltham, MA, USA).

#### 4.3.8. Nuclear Magnetic Resonance (NMR) 

The dried glucan and oligoglucan samples were weighed (30 mg each) and dissolved in 0.5 mL of heavy water in an NMR tube (Nuclear Magnetic Resonance Spectrometer, Bruker Avance NEO 600, Bruker Corporation, Billerica, MA, USA). ^1^H-NMR and ^13^C-NMR spectra were scanned at 25 °C with resonance frequencies of 600 MHz and 150 MHz, respectively [44].

### 4.4. Simulated Gastrointestinal Fermentation of Glucan and Oligoglucan

#### 4.4.1. In Vitro Fermentation Procedure

Fresh fecal samples were collected from three healthy volunteers within 1 h and stored immediately at −80 °C. The volunteers ranged in age from 23 to 28 years, were free of gastrointestinal disorders or diseases, and had not been treated with antibiotics or prebiotic supplements in the preceding 3 months. Samples were diluted with a sterilized diluent containing 1 L phosphate-buffered saline, 0.5 mg/L vitamin B1, and 0.5 mg/L vitamin B2 to produce a 10% fecal suspension. A 0.6 mL volume of each polysaccharide solution (0.5 g/mL) was mixed with 3 mL growth medium and fecal bacteria suspension with a total volume of 6.6 mL. The glucan and the oligoglucan were the experimental groups. Pure water and commercial oligosaccharide were the control groups. All groups were incubated at 37 °C in an anaerobic environment.

#### 4.4.2. Determination of pH, Strain Density, and Short-Chain Fatty Acids (SCFAs)

The gastrointestinal fermentation broth was collected at 0, 6, 12, 24, 36, and 48 h for determination. Strain density was determined by measuring the absorbance at 600 nm (OD_600_) using a spectrophotometer (UV-Visible Spectrophotometer, T6, Beijing General Analytical Instrument, Beijing, China). pH value was determined with a hand-held pH meter (HI98103, Hanna Instruments, Shanghai, China).

The fermentation solution was centrifuged (20 min, 8000 rpm) and filtered through a 0.22 µm membrane. HPLC (LC-20AT, Shimadzu Corporation, Kyoto, Japan) was performed using a device manufactured and equipped with the flame ionization detector to determine the SCFA content. Standard solutions of different concentrations of acetic acid, propionic acid, butyric acid, and valerate were configured. Acetonitrile was selected as mobile phase A and 0.025 (*v*/*v*) phosphoric acid aqueous solution was used as mobile phase B. They were applied in a 20:80 ratio of A:B. HPLC was performed at a flow rate of 1 mL/min, ultraviolet wavelength of 210 nm, C18 liquid chromatography column, column temperature of 30.0 °C, and sample size of 20 µL.

#### 4.4.3. Identification of Intestinal Flora in Fermentation Broth

Fermentation broth was collected and centrifuged (10,000 r/min, 4 °C, 20 min) 0 and 24 h. The sediments were stored at −80 °C for colony 16S rRNA gene [45].

### 4.5. Statistical Analysis

All experiments were repeated three times. Data were expressed as mean standard deviation (SD) values. All data were analyzed using Excel 2019, Origin 2021, SPSS 23.0, and one-way analysis of variance (ANOVA) to determine significant differences between samples. The significance level was set at *p* < 0.05.

## 5. Conclusions

Glucansucrase was obtained by the ultrafiltration centrifugation of the *L. mesenteroides* 6055 fermentation supernatant and indicated to be an extracellular and cell wall enzyme. This enzyme showed excellent viability at 20–35 °C and a neutral pH. The composition of the enzyme synthesized products (four oligoglucans: DP1–DP4), and the higher yields relative to the direct fermentation of the strain were confirmed through TLC and HPLC. The glucan and the oligoglucan property studies using SEM, zeta potential, apparent viscosity, and molecular weight analysis revealed physical differences. For example, glucan had a loose structure and fibrous form with a zeta potential value of −11.18 ± 0.15 mV, while oligoglucan had a denser texture and was dispersed in blocks with a zeta potential value of −18.83 ± 0.49 mV. XRD, FT-IR, and NMR analysis demonstrated that the glucan and the oligoglucan had similar crystallinity, functional groups, and structural characteristics. In vitro fecal fermentation indicated that the glucan and the oliglucans remarkably increased SCFAs contents and the abundance of microbial populations, such as *Bacteroides*, *Firmicutes*, *Verrucomicrobia*, and *Proteobacteria*. Based on these results, it can be concluded that both glucan and oligoglucan have excellent polysaccharide properties and potential prebiotic activity. This study provides new insights for polysaccharide synthesis and industrial/health applications. Further studies involving animal experiments are needed to assess the specific effects on certain diseases.

## Figures and Tables

**Table 1:** Molecular weight distribution of glucans and oligoglucans.

	0–2000	2000–10,000	>10,000	Mn (Da)	Mw (Da)	PD
Glucan	0	0	100%	844,190	1,117,981	1.32432
Oligoglucan	93.3%	6.483%	0.187%	725	3342	4.60966

## Data Availability

The data presented in this study are available upon request from the corresponding author.

## References

[1] Daba G.M., Elnahas M.O., Elkhateeb W.A. (2021). Contributions of exopolysaccharides from lactic acid bacteria as biotechnological tools in food, pharmaceutical, and medical applications. Int. J. Biol. Macromol..

[2] El-Deeb N.M., Yassin A.M., Al-Madboly L.A., El-Hawiet A. (2018). A novel purified *Lactobacillus acidophilus* 20079 exopolysaccharide, LA-EPS-20079, molecularly regulates both apoptotic and NF-kappaB inflammatory pathways in human colon cancer. Microb. Cell Fact..

[3] Angelin J., Kavitha M. (2020). Exopolysaccharides from probiotic bacteria and their health potential. Int. J. Biol. Macromol..

[4] Bhat B., Bajaj B.K. (2018). Hypocholesterolemic and bioactive potential of exopolysaccharide from a probiotic *Enterococcus faecium* K1 isolated from kalarei. Bioresour. Technol..

[5] Dertli E., Toker O.S., Durak M.Z., Yilmaz M.T., Tatlisu N.B., Sagdic O., Cankurt H. (2016). Development of a fermented ice-cream as influenced by in situ exopolysaccharide production: Rheological, molecular, microstructural and sensory characterization. Carbohydr. Polym..

[6] Niu M., Guo H., Cai J., Duan B., Chen Y., Meng X. (2023). Exopolysaccharide from *Bifidobacterium breve* alleviate dextran sulfate sodium-induced colitis in mice via inhibiting oxidative stress and regulating intestinal flora. Food Biosci..

[7] Wang Q., Jiang B., Wei M., He Y., Wang Y., Zhang Q., Wei H., Tao X. (2024). Antitumor effect of exopolysaccharide from *Lactiplantibacillus plantarum* WLPL09 on melanoma mice via regulating immunity and gut microbiota. Int. J. Biol. Macromol..

[8] Wang L., Gu Y., Zheng X., Zhang Y., Deng K., Wu T., Cheng H. (2021). Analysis of physicochemical properties of exopolysaccharide from *Leuconostoc mesenteroides* strain XR1 and its application in fermented milk. Lwt.

[9] Matsuzaki C., Takagaki C., Tomabechi Y., Forsberg L.S., Heiss C., Azadi P., Matsumoto K., Katoh T., Hosomi K., Kunisawa J. (2017). Structural characterization of the immunostimulatory exopolysaccharide produced by *Leuconostoc mesenteroides* strain NTM048. Carbohydr. Res..

[10] Miao M., Jia X., Hamaker B., Cui S., Jiang B., Huang C. (2016). Structure-prebiotic properties relationship for α-D-glucan from *Leuconostoc citreum* SK24.002. Food Hydrocoll..

[11] İspirli H., Bowman M.J., Skory C.D., Dertli E. (2021). Synthesis and characterization of cellobiose-derived oligosaccharides with Bifidogenic activity by glucansucrase E81. Food Biosci..

[12] Ko J.A., Nam S.H., Park J.Y., Wee Y., Kim D., Lee W.S., Ryu Y.B., Kim Y.M. (2016). Synthesis and characterization of glucosyl stevioside using Leuconostoc dextransucrase. Food Chem..

[13] Moon Y., Kim H., Kang C.G., Park C., Kim S.W., Kim D. (2022). Biochemical characterization of synthesized fisetin glucoside by dextransucrase from Leuconostoc mesenteroides NRRL B-1299CB4 with enhanced water solubility. Enzym. Microb. Technol..

[14] İspirli H., Yüzer M.O., Skory C., Colquhoun I.J., Sağdıç O., Dertli E. (2019). Characterization of a glucansucrase from *Lactobacillus reuteri* E81 and production of malto-oligosaccharides. Biocatal. Biotransform..

[15] Li Y., Liu Y., Cao C., Zhu X., Wang C., Wu R., Wu J. (2020). Extraction and biological activity of exopolysaccharide produced by *Leuconostoc mesenteroides* SN-8. Int. J. Biol. Macromol..

[16] Bisson G., Comuzzi C., Giordani E., Poletti D., Boaro M., Marino M. (2023). An exopolysaccharide from *Leuconostoc mesenteroides* showing interesting bioactivities versus foodborne microbial targets. Carbohydr. Polym..

[17] Wang B., Song Q., Zhao F., Zhang L., Han Y., Zhou Z. (2019). Isolation and characterization of dextran produced by *Lactobacillus sakei* L3 from Hubei sausage. Carbohydr. Polym..

[18] Bechtner J., Hassler V., Wefers D., Vogel R.F., Jakob F. (2021). Insights into extracellular dextran formation by *Liquorilactobacillus nagelii* TMW 1.1827 using secretomes obtained in the presence or absence of sucrose. Enzyme Microb. Technol..

[19] Liu L., Xu J., Na R., Du R., Ping W., Ge J., Zhao D. (2022). Purification, characterization and partial biological activities of exopolysaccharide produced by *Saccharomyces cerevisiae* Y3. Int. J. Biol. Macromol..

[20] Du R., Pei F., Kang J., Zhang W., Wang S., Ping W., Ling H., Ge J. (2022). Analysis of the structure and properties of dextran produced by *Weissella confusa*. Int. J. Biol. Macromol..

[21] Du R., Xing H., Yang Y., Jiang H., Zhou Z., Han Y. (2017). Optimization, purification and structural characterization of a dextran produced by *L. mesenteroides* isolated from Chinese sauerkraut. Carbohydr. Polym..

[22] Pan L., Zhou Z., Han Y. (2021). Exopolysaccharide from *Leuconostoc pseudomesenteroides* XG5 delay the onset of autoimmune diabetes by modulating gut microbiota and its metabolites SCFAs in NOD mice. J. Funct. Foods.

[23] Hu Y., Ganzle M.G. (2018). Effect of temperature on production of oligosaccharides and dextran by *Weissella cibaria* 10 M. Int. J. Food Microbiol..

[24] Tingirikari J.M., Kothari D., Shukla R., Goyal A. (2014). Structural and biocompatibility properties of dextran from Weissella cibaria JAG8 as food additive. Int. J. Food Sci. Nutr..

[25] Song L., Miao M., Jiang B., Xu T., Cui S., Zhang T. (2016). *Leuconostoc citreum* SK24.002 glucansucrase: Biochemical characterisation and de novo synthesis of α-glucan. Int. J. Biol. Macromol..

[26] Miao M., Ma Y., Jiang B., Cui S., Jin Z., Zhang T. (2017). Characterisations of *Lactobacillus reuteri* SK24.003 glucansucrase: Implications for α-gluco-poly- and oligosaccharides biosynthesis. Food Chem..

[27] Kim Y., Yeona M., Choia N., Chang Y., Jung M., Song J., Kim J. (2010). Purification and characterization of a novel glucansucrase from *Leuconostoc lactis* EG001. Microbiol. Res..

[28] Stecka K., Grzybowski R. (2000). The influence of pH and oxygen on the growth and probiotic activity of lactic acid bacteria. Food Biotechnol..

[29] Zeng M., Li N., Astmann T., Oh J.H., van Pijkeren J.P., Pan X. (2023). Facile and efficient chemical synthesis of gluco-oligosaccharides (GlcOS) with diverse glycosidic linkages as potential prebiotics to promote the growth of probiotic bacteria. Food Res. Int..

[30] Shukla R., Shukla S., Bivolarski V., Iliev I. (2011). Structural Characterization of Insoluble Dextran Produced by Leuconostoc mesenteroides NRRL B-1149 in the Presence of Maltose. Food Technol. Biotechnol..

[31] Yang Y., Peng Q., Guo Y., Han Y., Xiao H., Zhou Z. (2015). Isolation and characterization of dextran produced by Leuconostoc citreum NM105 from manchurian sauerkraut. Carbohydr. Polym..

[32] Cheng M., Qi J.R., Feng J.L., Cao J., Wang J.M., Yang X.Q. (2018). Pea soluble polysaccharides obtained from two enzyme-assisted extraction methods and their application as acidified milk drinks stabilizers. Food Res. Int..

[33] Jeddou K.B., Chaari F., Maktouf S., Nouri-Ellouz O., Helbert C.B., Ghorbel R.E. (2016). Structural, functional, and antioxidant properties of water-soluble polysaccharides from potatoes peels. Food Chem..

[34] Tang W., Zhou J., Xu Q., Dong M., Fan X., Rui X., Zhang Q., Chen X., Jiang M., Wu J. (2020). *In vitro* digestion and fermentation of released exopolysaccharides (r-EPS) from *Lactobacillus delbrueckii* ssp. *bulgaricus* SRFM-1. Carbohydr. Polym..

[35] Wang L., Li X., Wang B. (2018). Synthesis, characterization and antioxidant activity of selenium modified polysaccharides from *Hohenbuehelia serotina*. Int. J. Biol. Macromol..

[36] Nakamura N., Hamazaki T., Jokaji H., Minami S., Kobayashi M. (1998). Effect of HMG-CoA reductase inhibitors on plasma polyunsaturated fatty acid concentrations in patients with hyperlipidemia. Int. J. Clin. Lab. Res..

[37] Schilderink R., Verseijden C., Seppen J., Muncan V., van den Brink G.R., Lambers T.T., van Tol E.A., de Jonge W.J. (2016). The SCFA butyrate stimulates the epithelial production of retinoic acid via inhibition of epithelial HDAC. Am. J. Physiol. Gastrointest. Liver Physiol..

[38] Ding W., Fang Q., Zhou W., Ping Q., Xiao Y., Wang Z. (2023). Performance and mechanism of sodium citrate pretreatment to promote waste activated sludge disintegration and short-chain fatty acid production during anaerobic fermentation. J. Environ. Chem. Eng..

[39] Du H., Zhao A., Wang Q., Yang X., Ren D. (2020). Supplementation of Inulin with Various Degree of Polymerization Ameliorates Liver Injury and Gut Microbiota Dysbiosis in High Fat-Fed Obese Mice. J. Agric. Food Chem..

[40] Ghotaslou R., Nabizadeh E., Memar M.Y., Law W.M.H., Ozma M.A., Abdi M., Yekani M., Kadkhoda H., Hosseinpour R., Bafadam S. (2023). The metabolic, protective, and immune functions of *Akkermansia muciniphila*. Microbiol. Res..

[41] Hiippala K., Jouhten H., Ronkainen A., Hartikainen A., Kainulainen V., Jalanka J., Satokari R. (2018). The Potential of Gut Commensals in Reinforcing Intestinal Barrier Function and Alleviating Inflammation. Nutrients.

[42] Ding Y., Yan Y., Peng Y., Chen D., Mi J., Lu L., Luo Q., Li X., Zeng X., Cao Y. (2019). In vitro digestion under simulated saliva, gastric and small intestinal conditions and fermentation by human gut microbiota of polysaccharides from the fruits of *Lycium barbarum*. Int. J. Biol. Macromol..

[43] Ma Y., Jiang S., Zeng M. (2021). In vitro simulated digestion and fermentation characteristics of polysaccharide from oyster (*Crassostrea gigas*), and its effects on the gut microbiota. Food Res. Int..

[44] Guo Y., Wang L., Li L., Zhang Z., Zhang J., Zhang J., Wang J. (2022). Characterization of polysaccharide fractions from *Allii macrostemonis bulbus* and assessment of their antioxidant. Lwt.

[45] Liang D., Li N., Dai X., Zhang H., Hu H. (2021). Effects of different types of potato resistant starches on intestinal microbiota and short-chain fatty acids under in vitro fermentation. Int. J. Food Sci. Technol..

